# Penalty feet positioning rule modification and laterality effect on soccer goalkeepers’ diving kinematics

**DOI:** 10.1038/s41598-022-21508-6

**Published:** 2022-11-02

**Authors:** Rafael Luiz Martins Monteiro, Bruno Luiz Souza Bedo, Pedro Henrique Martins Monteiro, Felipe dos Santos Pinto de Andrade, Felipe Arruda Moura, Sergio Augusto Cunha, Ricardo da Silva Torres, Daniel Memmert, Paulo Roberto Pereira Santiago

**Affiliations:** 1 Biomechanics and Motor Control Laboratory, School of Physical Education and Sport of Ribeirão Preto, University of São Paulo (USP), Ribeirão Preto, 14040-907 Brazil; 2 Program in Rehabilitation and Functional Performance, Ribeirão Preto Medical School, University of São Paulo (USP), Ribeirão Preto, 14049-900 Brazil; 3 School of Physical Education and Sport, University of São Paulo (USP), São Paulo, 05508-030 Brazil; 4 Laboratory of Applied Biomechanics, Department of Sport Sciences, Centre of Physical Education and Sport, State University of Londrina, Londrina, 86057‑970 Brazil; 5 Department of Sport Sciences, University of Campinas, Campinas, Brazil; 6 Department of ICT and Natural Sciences, NTNU – Norwegian University of Science and Technology, Aalesund, Norway; 7 Institute of Exercise Training and Sport Informatics, German Sport University Cologne, Am Sportpark Müngersdorf 6, 50933 Cologne, Germany

**Keywords:** Musculoskeletal system, Biomedical engineering, Biological physics, Motor control

## Abstract

In 2019, a new rule was applied in soccer. It allows the goalkeeper to have only one foot or part of it on the goal line when the kicker hits the ball, unlike the previous rule that determined the goalkeeper should have both feet on the line. The purpose of the present study was to analyze how the change in the rule and the lower limbs laterality influences on the diving save kinematic performance in penalties. Six goalkeepers, two professionals and four amateurs, performed a total of 20 dives in the laboratory and had their force and impulse exerted by the lower limb and displacement/velocity data from the center of body mass collected through force plates and kinematic analysis. The side preference was collected through an inventory. The results showed that goalkeepers dive further (p < 0.001) and faster (p < 0.001) when diving according to the new rule. Dives for the non-dominant side presented higher values than the trials for the dominant side in mediolateral (p = 0.02) and resultant (p = 0.03) displacements. Concluding, the goalkeepers performed better with the new rule in the analyzed variables and the lower limb preference has influenced only the mediolateral and resultant displacement.

## Introduction

World cup goal scoring pattern analysis over time (1998–2018) indicates that the number of goals scored resultant from set play (corner kick, direct free kick, free kick assist, penalty kick, and throw- in assist) represents 29.33 ± 5.57% of the goals scored^1–4^. When other soccer championships around the world are analysed (i.e. Champions league 2016/2017–2019/2020 and Copa libertadores 2017–2020) the goals from set play represents 21.88 ± 9.96% of the goals scored^5^. The penalty represents 25.71–47.56% of the set play goals^1,3,6,7^, however these numbers tend to grow with the use of video assistant referee (VAR)^8^. In other words, the penalty is and tends to be more decisive in determining the winner of matches and championships.

The penalty was officially introduced in the soccer rules by the International Football Association Board (IFAB) in 1891. In its first version the penalty could be kicked from any point 11 m from the goal line and the goalkeeper could stay anywhere within 5.5 m from the goal^9^. Only in 1902 the penalty area and spot were introduced^10^. In 1905 the goalkeeper positioning rule was changed and he/she must stay in the goal line being permitted to move the feet along it^11^. In 1930 the goalkeeper was forbidden to move the feet until the penalty kick was taken^12^. Only in 1997 this rule changed and the goalkeeper was permitted to move the feet on the goal line again^13^. As it is possible to notice the soccer penalty rules changed frequently along the history and keeps changing.

On March 13, 2019, the IFAB officialized new changes in soccer rules, with the application starting on June 1, 2019. Among them, a new goalkeeper's feet positioning rule was determined in the penalty. In the old rule (OR), the goalkeeper must keep the feet on the goal line until the kicker hits the ball. In contrast, in the new rule (law 14 of the document ``Laws of the game 2019/20 changes and clarifications'')^14^ (NR), the goalkeeper may keep only part of one foot on the goal line at the moment the kicker hits the ball. This rule change demands research to verify its impact on the goalkeeper's diving kinematics in penalties.

Several studies have investigated what strategies goalkeepers can benefit from in penalty situations^15–22^. Being in movement has shown to be a good strategy to distract the kicker and decrease his/her performance^15,16^. Another interesting strategy is the goalkeeper to position himself out of the goal center, even if it is in an apparently unperceptive way, because it can induce the kicker to direct the ball to the goal side with more space^17^. Also, an association between ball’s and goalkeeper’s velocity on the penalty concluded that goalkeepers must anticipate to reach the majority of balls in the corners of the goal^18^. Studies of the goalkeeper’s visual dynamics have attested that for successful anticipation, a kick direction prediction is necessary^19^ and that goalkeepers present better reaction time to predict the kick direction when compared with outfield players^20^.

In the biomechanics area, the subject has already been explored as well. A study investigated six elite goalkeepers in penalty diving save situations and concluded that the thorax and pelvis movements caused the asymmetries between the diving sides^23^. Other studies with goalkeeper’s kinematic analysis concluded that the contralateral lower limb has a more significant contribution to mediolateral velocity, and the diving save preparatory feet position is important for determining the dive performance^24,25^.

The relation between laterality and performance in penalty goalkeeper’s diving save has already been investigated. Contradictory results were found between different studies. Some found greater center of body mass (CM) velocity in the contact with the ball when diving to the dominant lower limb (DLL) side^23^ while others did not find differences in the CM velocity between the diving sides^24^. Some found that in dives to the non-dominant lower limb (NDLL) side, the CM reached highest height^23^ but others did not find differences in the diving CM displacement and attested that the NDLL side had a bigger variability on the consecutive dives when compared to the DLL side^26^. All of this elucidates that the scientific literature lacks more studies to investigate how laterality influences on the kinematics of soccer goalkeepers' diving save in penalties.

In summary, several studies have investigated the goalkeeper’s training for the defender to succeed in their function on decisive moments as the penalty. Due to the change of rule and considering the current literature state, researchers must understand how this change will affect the kinematics of the goalkeeper's dive in the penalty. This study will help trainers to understand: (I) if it is worth orienting their athletes to use the frontal step allowed by the new rule; (II) how occurs the force generation in the diving impulse in penalty diving save performance; and (III) how laterality influences on the goalkeeper diving save kinematic. Therefore, the present study aimed to analyze the goalkeeper's dive in soccer penalties, comparing the goalkeeper's feet positioning rules and laterality effect in the performance. Our initial hypotheses were that (I) the NR trials would present greater values in most variables; (II) there would be no laterality effects on the dive variables.

## Methods

### Participants

Six soccer goalkeepers (23.68 ± 3.81 years old; 81.6 ± 13.02 kg; 1.85 ± 0.05 m; 15 ± 4.32 years of experience in the position; training frequency = 4.17 ± 2.24 times a week) participated in this study. Two of the participants were participating in Brazilian youth official championships and the rest was playing in amateur and/or university level championships. Among the volunteers, only one was left-footed at the lower limbs lateral preference. The selected athletes did not present any sports injuries in the past two months which resulted in the absence of training for a period equal to or greater than fifteen days. Studies involving goalkeeper diving kinematic analysis tends to have a low number of participants. The mean number of volunteers in this kind of research is^23–29^ 6.33 ± 3.5. The experiments were performed within the ethical standards set out in Resolution 466/12 of the National Health Council of 12/12/2012 (BRASIL, 2012) and the Resolution of Helsinki (2001). The School of Physical Education and Sport of Ribeirão Preto ethics committee (5659) approved all the experimental procedures (CAAE: 24268719.0.0000.5659). Written consent was obtained from the participants or their legal guardian(s).

### Instruments

The participants realized the collection with 39 reflexive circular markers of 24 mm attached to the body. A standard Vicon's protocol, the full-body modeling with plug-in gait^30^, was utilized for the markers’ locations. For three-dimensional motion data collection, a system of ten infrared cameras was used, adjusted in an acquisition frequency of 400 Hz (Vicon, Oxford, UK). A stick marked with known distances was used to calibrate the system. The errors and lens distortions were adjusted using the software Nexus.

Two force plates (40 × 60 cm, Bertec, Columbus, USA), adjusted in an acquisition frequency of 2000 Hz, were used to collect the force and impulse exerted by the lower limb ipsilateral to the side of the dive. The Global Lateral Preference Inventory (IPLAG)^31^ was used to identify the volunteer's lower limbs' lateral preference.

### Experimental procedures

Initially, the volunteers received instructions about the collection procedures. Then, they filled out the IPLAG for the lower limbs lateral preference identification. Before starting the execution of the dives, each participant realized a warm-up conducted by the researchers following some principles proposed by the literature^32^. They completed a sequence of exercises with 5–10 min of duration at a lower intensity. The warm-up final part was the volunteers familiarization with the penalty simulation through the execution of four dives to the right (two with the OR and two with the NR) and four to the left (the same number with each rule).

The goalkeeper was positioned in a previously demarcated location for the test performance. If the goalkeeper would perform the diving save according to the OR, he was instructed to position the foot ipsilateral to the dive side on the force plate and could only take impulse with a horizontal lower limb movement in a way the feet don't leave the goal line in the diving impulsion. When the volunteer was going to execute the dive according to the NR, he was instructed to position himself in a way that the ipsilateral foot hits the force plate during the diving movement and was instructed to frontally project the foot in the impulsion in a way he would have only one foot or part of it in the goal line in the moment the kicker hits the ball.

**Figure 1:**
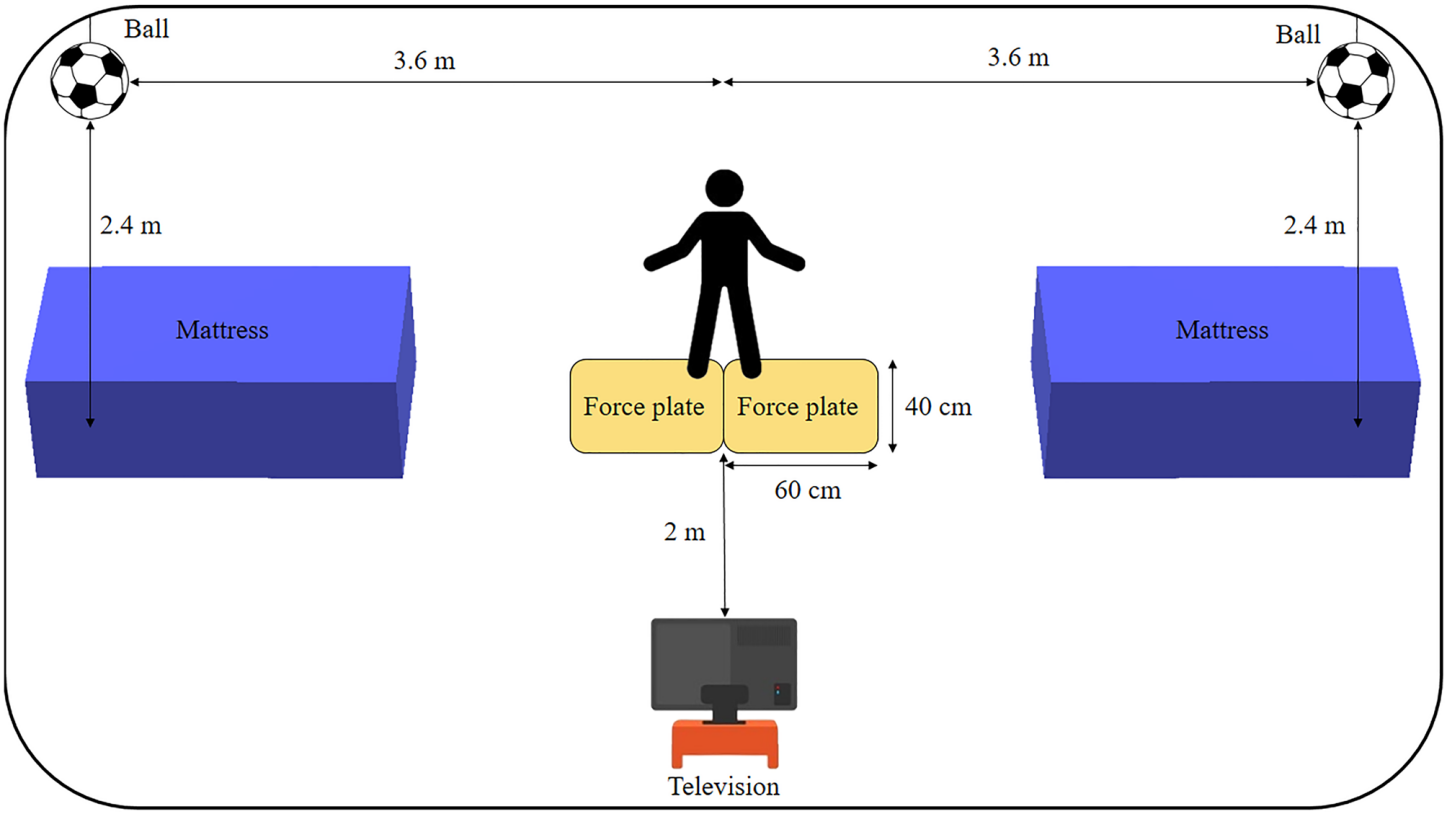
Experimental setup of the laboratory at the moment of the collection.

The goalkeeper was warned previously regarding the side he would dive and if it would be according to the OR or NR. The collection started when the researchers executed a soccer penalty kick video seen by the goalkeeper’s vision. The videos contained only kicks in which the ball trajectory passed a lateral distance of 3.5 m from the goal center and a height of 1.6 m. The goalkeeper was guided to initiate the movement according to the penalty kick of the video. Two balls were hung in the laboratory in the distance that the balls would have crossed the goal line to serve as a target to the defense simulated in the dive (Fig. 1). Despite only 9.4% of the penalties are kicked in the goal upper corners^33^, this place was chosen because it is the point that demands the greatest horizontal and vertical displacement from the goalkeepers in penalty diving saves. Mattresses were placed in the laboratory to cushion the fall impact.

The volunteers performed a total of 20 dives, being 10 with each feet positioning rule and an equal number of dives for each side. There were 10 dives to the right, 5 with the OR and NR, and 10 to the left with the same number of trials with each rule. The order of the dive executions was random so this factor does not influence the final result. The recovery time between the trials was equal to or greater than 90 s.

### Data analysis

To determine laterality, the IPLAG results were observed. The lateral preference of the lower limbs was extracted. The IPLAG provides a numeric scale, in which: 1 = strongly left-footed; 2 = moderate left-footed; 3 = ambidextrous or without preference; 4 = moderate right-footed; 5 = strongly right-footed. However, to separate the diving saves between the dominant lower limb side and non-dominant lower limb side, the volunteers classified as 1 and 2 were grouped in left-footed and the ones classified as 4 and 5 formed the right-footed group. No one was classified in category 3.

The data processing was similar to the one utilized in previous study^29^. For diving performance variables analysis, three-dimensional CM data and the values collected by the force plate were used. The variables calculations were made as described: (I) Three-dimensional diving displacement was given by the root of the sum of squares of the displacement in each of the three axes concerning the goalkeeper (X—transversal; Y—sagittal; Z—longitudinal). The displacement beginning was set as the CM Z-axis minor point and the end as the CM Z-axis greater point; (II) The dive average velocity was determined through the diving resultant displacement divided by the time spent on the displacement; (III) Peak velocity was calculated by the greater value of displacement over time between the lower and greater height of the CM in the rising phase of the dive; (IV) Peak force exerted by the lower limb ipsilateral to the dive side normalized by the weight was obtained by identifying the greater value of force divided by the weight; and (V) Impulse exerted by the lower limb ipsilateral to the dive side normalized by the weight was determined through the trapezoidal integral of the force x time graph only at the moment of the impulse divided by the weight.

The CM three-dimensional coordinates were calculated using the software Nexus since we used a markers’ location standard protocol. In cases where the cameras did not capture the essential markers to the CM calculation in the final frames, the anatomical point was transferred to a non-essential marker of the same corporal segment. When this procedure was not possible, the trial was discarded. A total of 120 dives were processed, 16 were discarded due to the data capture problems described. Therefore, 104 dives were considered in the analysis. It was developed a custom routine on Python 3 to process and obtain the variables of interest. The raw data was filtered with the digital Butterworth filter of fourth-order with cut-off frequency of 10 Hz obtained by residual analysis^34^.

### Statistical analysis

For presenting the descriptive results, average ± standard deviation (SD) was used. The data normality was confirmed using the Shapiro–Wilk test. The Student’s paired t-test indicated the performance differences between the rules and between the sides of the dive lateral preference. The Cohen’s d was used to report the effect size of the presented variables (0.2–0.3 small, 0.5–0.8 medium, > 0.8 large)^35^. In all cases, the significance level was p ≤ 0.05. The analysis were conducted in the software SPSS (IBM Corp. Released 2012. IBM SPSS Statistics for Windows, Version 21.0. Armonk, NY: IBM Corp.).

## Results

**Figure 2:**
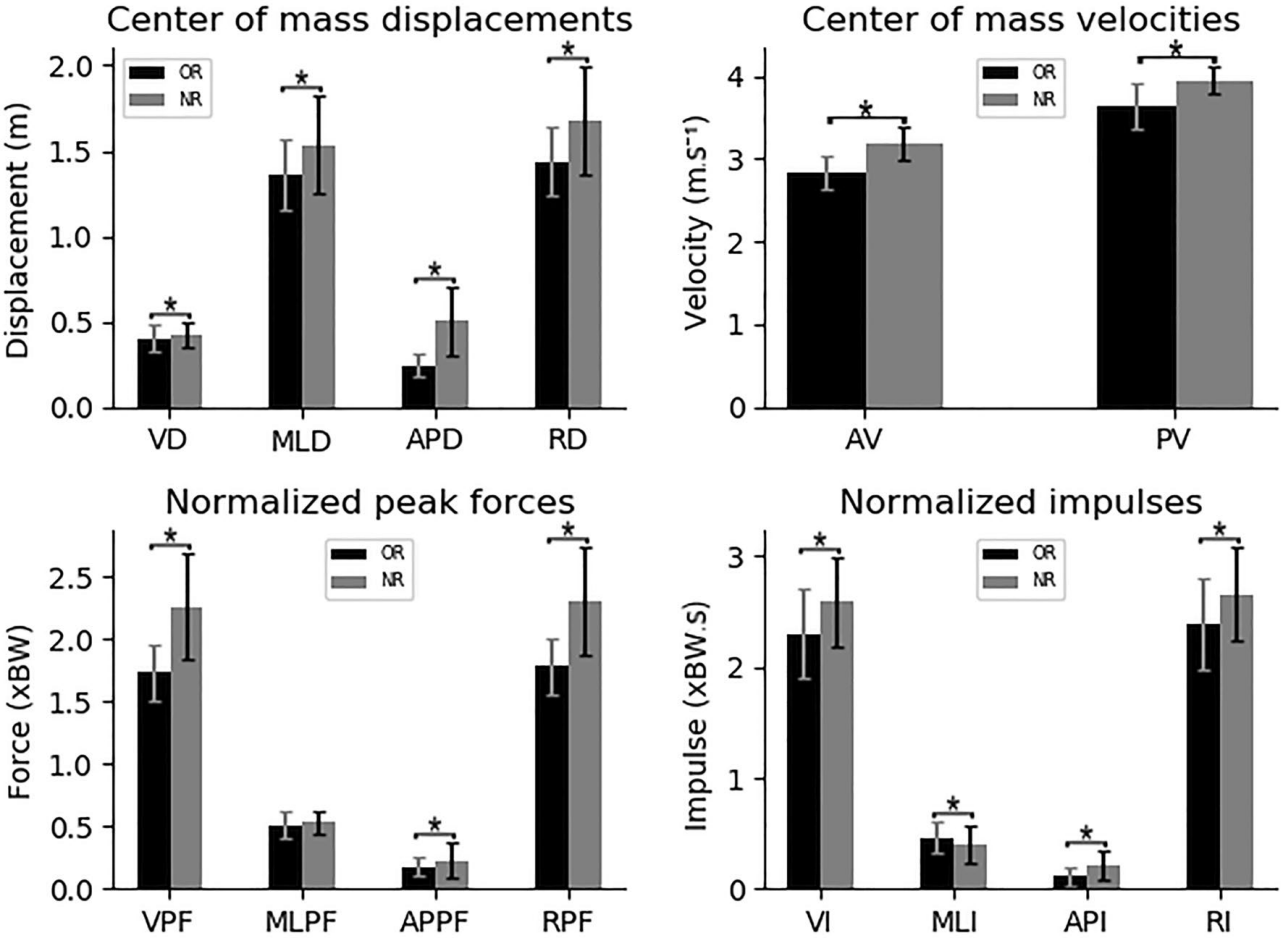
Average and standard deviation of the dive variables with the trials divided according to the rules independently of the diving side. *p < 0.05. OR = trials with the old rule; NR, trials with the new rule; VD, vertical displacement; MLD, mediolateral displacement; APD, anteroposterior displacement; RD, resultant displacement; AV, average velocity; PV, peak velocity; VPF, vertical peak force; MLPF, mediolateral peak force; APPF, anteroposterior peak force; RPF, resultant peak force; VI, vertical impulse; MLI, mediolateral impulse; API, anteroposterior impulse; RI, resultant impulse; m, meters; s, seconds; xBW, times body weight.

In the first analysis, the dives were divided in relation to the rules independently of the diving side (Fig. 2). The trials with the NR presented greater values in relation to the ones with the OR in the following variables: vertical displacement (VD) (0.4 ± 0.77–0.425 ± 0.0728 m, p = 0.03, d = 0.28), mediolateral displacement (MLD) (1.36 ± 0.202–1.536 ± 0.293 m, p < 0.001, d = 0.56), anteroposterior displacement (APD) (0.247 ± 0.069–0.503 ± 0.197 m, p < 0.001, d = 1.84), resultant displacement (RD) (1.44 ± 0.2–1.679 ± 0.324 m, p < 0.001, d = 0.87), average velocity (AV) (2.836 ± 0.2—3.179 ± 0.203 m s^−1^, p < 0.001, d = 1.74), peak velocity (PV) (3.643 ± 0.276–3.953 ± 0.177 m s^−1^, p < 0.001, d = 1.37), vertical peak force (VPF) (1.73 ± 0.223–2.261 ± 0.431 xBW, p < 0.001, d = 1.55), anteroposterior peak force (APPF) (1.793 ± 0.075–0.225 ± 0.142 xBW, p = 0.04, d = 0.45), resultant peak force (RPF) (1.782 ± 0.22–2.303 ± 0.435 xBW, p < 0.001, d = 1.53), vertical impulse (VI) (2.305 ± 0.386–2.588 ± 0.415xBW.s, p < 0.001, d = 0.70), anteroposterior impulse (API) (0.111 ± 0.078–0.213 ± 0.129 xBW.s, p < 0.001, d = 1.01) and resultant impulse (RI) (2.384 ± 0.401–2.661 ± 0.426 xBW.s, p < 0.001, d = 0.68). However, the OR trials presented only one variable with greater values compared with the NR trials, the mediolateral impulse (MLI) (0.398 ± 0.176–0.464 ± 0.136 xBW.s, p = 0.02, d = 0.46).

**Figure 3:**
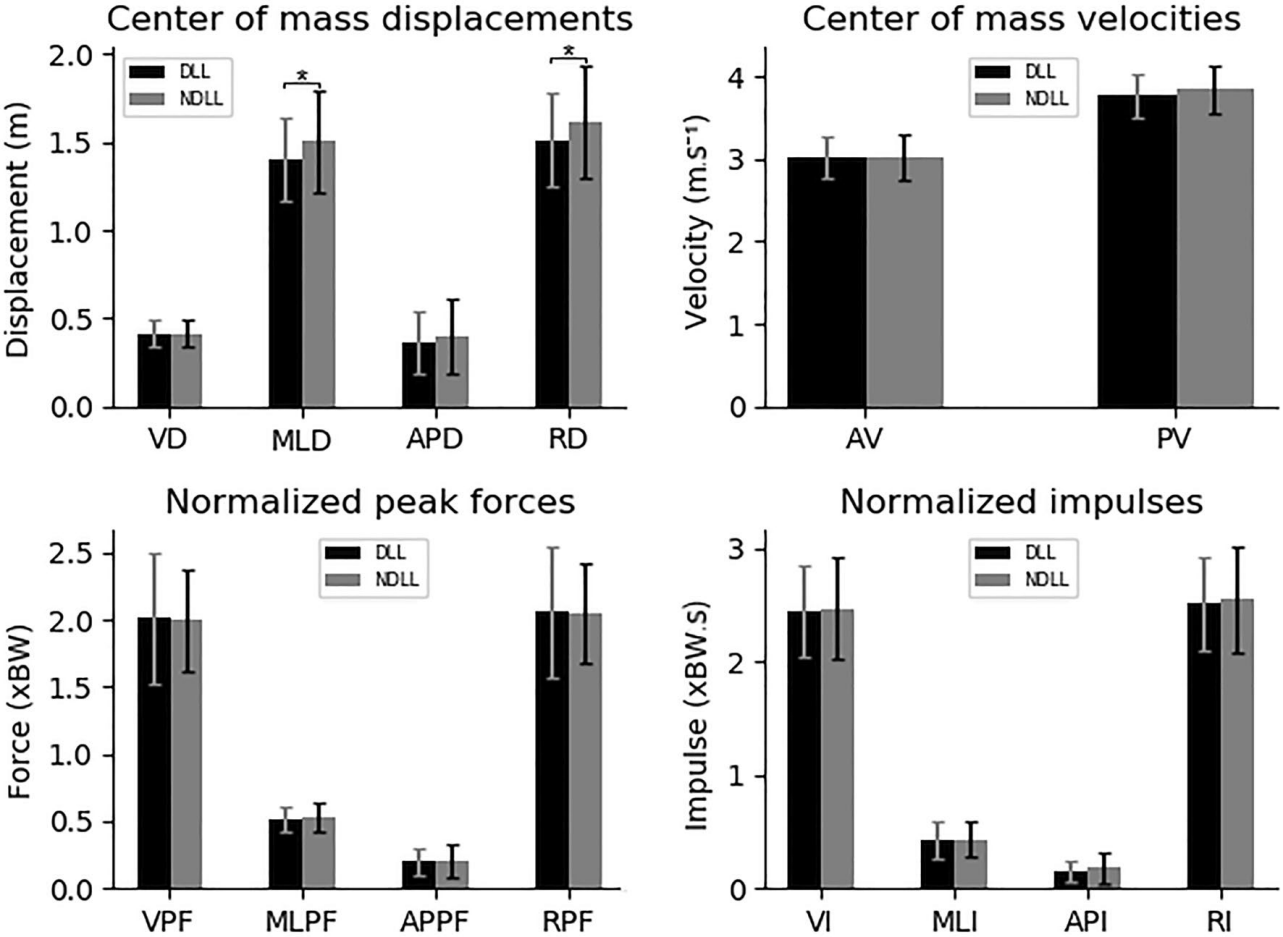
Average and standard deviation of the dive variables with the trials divided according to the lower limbs laterality independently of the rule the trials were performed. *p < 0.05. DLL, trials to the side of the dominant lower limb; NDLL, trials to the side of the non-dominant lower limb; VD, vertical displacement; MLD, mediolateral displacement; APD, anteroposterior displacement; RD, resultant displacement; AV, average velocity; PV, peak velocity; VPF, vertical peak force; MLPF, mediolateral peak force; APPF, anteroposterior peak force; RPF, resultant peak force; VI, vertical impulse; MLI, mediolateral impulse; API, anteroposterior impulse; RI, resultant impulse; m, meters; s, seconds; xBW, times body weight.

In the second analysis, the dives were divided according to the lateral dominance of the lower limbs independently of the rule the trials were performed (Fig. 3). The trials to the side of the NDLL presented greater values in comparison to the trials to the side of the DLL in only the following variables: MLD (1.399 ± 0.24–1.497 ± 0.282 m, p = 0.02, d = 0.38) and RD (1.512 ± 0.266–1.609 ± 0.312 m, p = 0.03, d = 0.31).

**Figure 4:**
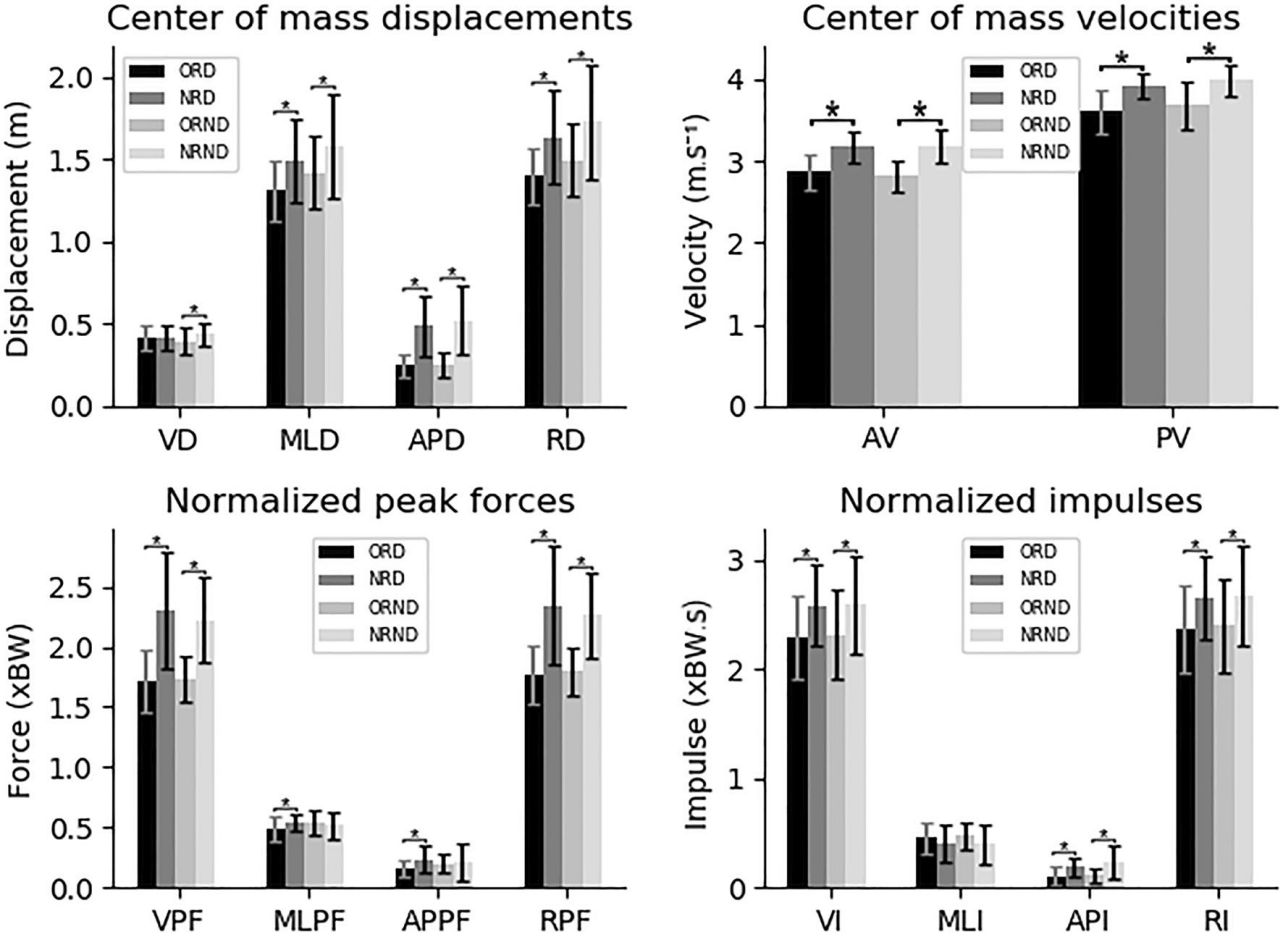
Average and standard deviation of the dive variables with the trials divided according to the rules and lower limbs laterality, comparing the old and new rule in each diving side. *p < 0.05. ORD, trials with the old rule to the side of the dominant lower limb; NRD, trials with the new rule to the side of the dominant lower limb; ORND, trials with the old rule to the side of the non-dominant lower limb; NRND, trials with the new rule to the side of the non-dominant lower limb; VD, vertical displacement; MLD, mediolateral displacement; APD, anteroposterior displacement; RD, resultant displacement; AV, average velocity; PV, peak velocity; VPF, vertical peak force; MLPF, mediolateral peak force; APPF, anteroposterior peak force; RPF, resultant peak force; VI, vertical impulse; MLI, mediolateral impulse; API, anteroposterior impulse; RI, resultant impulse; m, meters; s, seconds; xBW, times body weight.

In the last analysis, the dives were divided according to the rules and lower limbs laterality, comparing the trials with the OR and NR in each diving side (Fig. 4). The dives with the NR to the side of the DLL (NRD) presented greater values in relation with the OR to this same side (ORD) in the following variables of the CM displacement, velocity, DLL normalized force and impulse: MLD (1.3 ± 0.183–1.492 ± 0.257 m, p < 0.001, d = 0.87), APD (0.246 ± 0.069–0.485 ± 0.181 m, p < 0.001, d = 1.78), RD (1.394 ± 0.179–1.63 ± 0.288 m, p < 0.001, d = 1.07), AV (2.862 ± 0.225–3.17 ± 0.2 m s^−1^, p < 0.001, d = 1.42), PV (3.608 ± 0.269—3.919 ± 0.144 m s^−1^, p < 0.001, d = 1.48), VPF (1.722 ± 0.262–2.302 ± 0.492 times body weight (xBW), p < 0.001, d = 1.47), mediolateral peak force (MLPF) (0.487 ± 0.108–0.541 ± 0.07 xBW, p = 0.03, d = 0.69), APPF (0.159 ± 0.072–0.237 ± 0.116 xBW, p = 0.005, d = 0.86), RPF (1.768 ± 0.255–2.345 ± 0.503 xBW, p < 0.001, d = 0.90), VI (2.292 ± 0.389–2.581 ± 0.381 xBW.s, p = 0.002, d = 0.76), API (0.108 ± 0.093–0.189 ± 0.085 xBW.s, p = 0.002, d = 0.93) and RI (2.399 ± 0.405–2.652 ± 0.388 xBW.s, p = 0.002, d = 0.74).

The dives with the NR to the side of the NDLL (NRND) presented greater values in comparison to the dives with the OR to this same side (ODND) in the following variables: VD (0.391 ± 0.774–0.433 ± 0.703 m, p = 0.02, d = 0.53), MLD (1.414 ± 0.209–1.58 ± 0.324 m, p = 0.02, d = 0.63), APD (0.247 ± 0.698–0.52 ± 0.213 m, p < 0.001, d = 1.81), RD (1.491 ± 0.21–1.727 ± 0.355 m, p = 0.003, d = 0.79), AV (2.811 ± 0.171–3.189 ± 0.208 m s^−1^, p < 0.001, d = 1.99), PV (3.677 ± 0.284–3.987 ± 0.202 m s^−1^, p < 0.001, d = 1.27), VPF (1.739 ± 0.181–2.22 ± 0.364 xBW, p < 0.001, d = 1.65), RPF (1.796 ± 0.182–2.261 ± 0.361 xBW, p < 0.001, d = 1.65), VI (2.319 ± 0.39–2.594 ± 0.454 xBW.s, p = 0.02, d = 0.66), API (0.113 ± 0.06–0.238 ± 0.16xBW.s, p = 0.001, d = 0.99) and RI (2.399 ± 0.404–2.67 ± 0.468 xBW.s, p = 0.03, d = 0.85).

## Discussion

This study aimed to compare the goalkeeper’s performance in the dives with the OR and NR in the penalty and describe the effect of laterality on this phenomenon. About the performance, this research demonstrated that the variables of displacement and velocity were all greater when the dive was realized with the NR. In other words, the goalkeeper dives farther and faster when he can project a foot forward in the diving impulse. Diving faster permits the goalkeeper to wait more time before jumping, giving him more time to try to predict the side the ball will be kicked rising the chance to guess it^36–38^.

When analyzing the peak force and impulse of the lower limb ipsilateral to the diving side, it is evident that these variables are greater for the vertical, anteroposterior and resultant direction. These findings demonstrate that the frontal foot projection movement pattern allowed by the NR interferes and benefits the force and impulse applied with the ipsilateral lower limb. However, in the mediolateral direction, it is observed that there was no difference in the peak force and the dives with the OR presented greater values of impulse. These results show a greater ipsilateral lower limb dependence on the MLI production in this rule, which was ineffective due to the lower MLD presented.

These results related to the MLI and MLPF demonstrate that the force and impulse in the mediolateral direction are generated mainly by the contralateral lower limb. This is even more evident when it is observed that in the NR trials, even though the MLI is lower and the MLPF does not show any difference in comparison to the OR, MLD is higher, which allows the conclusion that the impulse to this greater displacement can only have come from the contralateral lower limb, which corroborates with other's findings^24^.

When observing the results regarding the lower limbs lateral dominance, it can be noted that most of the variables do not show a difference between the trials to the DLL and NDLL sides. The low asymmetry may be due to soccer game requirements of similar side-to-side and hamstrings to quadriceps relationships regardless of position^39,40^. The goalkeepers' specific trainings (i.e. explosive lateral movements,dives, and vertical jumps)^41^ also influence on this low asymmetry and provides them a greater quadriceps and hamstrings concentric strength for the preferred and non preferred legs compared with almost all other positions^39^.

However, it was still possible to observe the effects of the lateral dominance, although presenting a small effect size, the MLD and consequently the RD had greater values when the trial was made to the side of the NDLL in comparison to the DLL. Our hypothesis for explaining these results is that the goalkeepers that participated in this study have a stronger DLL, because when they dive to the NDLL side, the main responsible for the MLD generation is the DLL. Further studies need to be done to investigate this question. Better turning direction for the NDLL side in 90 degrees change of direction tasks has already been found in the majority of soccer players in a sample with 94 young highly trained male soccer players, showing the importance of the DLL in this kind of task^42^.

Other studies were performed to investigate the laterality effect on soccer goalkeeper penalty diving save^23,24,26^. Differently from our results, they found that the CM had greater velocity in the contact with the ball when diving to the side of the DLL and reached the highest height to the side of the NDLL^23^. While a previous study did not find differences in the MLD between the diving sides, it reported similar results to the present study regarding the VD. It is essential to highlight that the study evaluated only one goalkeeper, and no training level was reported^26^. Another research used specialized goalkeepers and found similar results regarding laterality, i.e., it did not find differences in the CM velocity between the dives to both sides^24^.

The limitations of this study were: (I) the ball being static at the moment of the dive; (II) the collection having been carried out inside a laboratory setting; (III) the necessity of the goalkeeper to step on the force plate to perform the dive. These factors may have taken the collection away from what happens in real penalty situations but is aligned with the experimental procedures used in other diving save kinematic analysis studies^23–29^. The authors suggest that future studies should be carried out on the field and with balls in motion. An interesting alternative for markerless motion capture for in field data collection is the use of trained artificial neural network for automatic identification of joints and anatomical references, such as OpenPose^43–46^. The 2D data acquired by the OpenPose can be transformed into 3D global coordinates using the 3D-DLT method (direct linear transformation). We also emphasize that the literature lacks biomechanical studies analyzing the dynamics of soccer goalkeeper's dives and the relationship between the goalkeeper and lateral dominance.

Despite the limitations of this study, biomechanical analysis can establish themselves as important ways of soccer goalkeeper dive evaluation. It permits precise inferences in the analyzed variables. Besides that, they are an important tool for understanding the diving process, indicating the athletes' deficiencies and limitations concerning laterality, force, and impulse generation. All of this can contribute to improving the soccer goalkeepers training.

### Practical application

Trainers can use our results to improve their goalkeepers' diving save velocity and displacement by instructing their athletes to frontally project one foot in the diving impulse moment. They can also introduce contralateral exercises to their goalkeepers' training routine in an attempt to improve their diving save mediolateral impulse generation. Furthermore, trainers could use biomechanical analysis to accurately investigate their goalkeepers' diving save asymmetries, because there could be differences for asymmetry results when using general strength (i.e. isokinetic muscular strength of knee joint flexion and extension) and specific task tests for evaluation^47^.

## Conclusions

The soccer goalkeepers dived farther and faster when they used the frontal foot projection allowed by the new feet positioning penalty rule. The lateral dominance influenced only the mediolateral displacement and resultant displacement in penalty diving saves, presenting greater values to the side of the non-dominant lower limb. Also, it was possible to identify that the contralateral lower limb had major participation in the mediolateral impulse generation.

## Data Availability

The datasets generated and analyzed during the current study and the code for the data analysis pipeline performed are available on figshare^48^ (https://doi.org/10.6084/m9.figshare.19059086.v1) and https://github.com/rafaellmmonteiro/DataDivingGoalkeepers (As of Jan. 2021).
